# Supplementary material for: Discovery of Carbonic Anhydrase 9 as a Novel CLEC2 Ligand in a Cellular Interactome Screen
Source: Cells. 2024 Dec 17;13(24):2083. doi: 10.3390/cells13242083 (PMC11674933; doi:10.3390/cells13242083)
Supplement: Supplementary file 1 [file cells-13-02083-s001.zip › cells-3341200-Supplementary .pdf]

Supplemental Data

Plasmid library

Constructs of the plasmid library were mainly synthesized by Twist Biosciences. Some constructs were ordered from Thermo Fisher Scientific GeneArt.

Constructs which were N terminally tagged with a mutPlGF tag received a signaling sequence of CD52. For coding DNA (cDNA), amino acid sequences were translated with codon optimization for expression in human. Kozak sequence and cloning sites (NotI/BamHI or NotI/XbaI) for construct insertion into vector backbone were added to cDNAs. cDNA synthesis, cloning into expression vectors, and plasmid preparation was performed by Twist Biosciences or Thermo Fisher Scientific GeneArt. Constructs were either cloned into pTwist CMV vectors holding the respective tag sequence (Twist Bioscience) or cloned as full-length expression constructs into empty pOptiVec plasmids (Thermo Fisher Scientific GeneArt).

Plasmid constructs

Plasmids that were designed in addition to the plasmid library were synthesized by Thermo Fisher Scientific GeneArt using codon optimization, Kozak sequence and restriction sites (XbaI/NotI) for subcloning into an expression vector backbone (pOptiVec). Insert protein sequences are listed in Table S1.

Plasmid constructs were transiently transfected in HEK-293T for expression into conditioned media. For G250 constructs, heavy (Fc(dead) or Fc removed) and light chain were transiently co-transfected in a 1:1 ratio. Three days post transfection conditioned media was centrifuged for 5min at 300 g and supernatants were collected.

Supplemental Table S1. Protein sequence of constructs used for binding experiments or cell line generation

Name	protein sequence
CA9-ectodomain (full-length) with mutPLGF tag and StrepII tag sequence	MKRFLFLLLTISLLVMVQIQTGLSQRLPRMQEDSPLGGGSSGEDDPLGEEDLPSEEDSPREEDPPGEEDLPGEEDLPGEEDLPEVKPKSEEEGSLKLEDLPTVEAPGDPQEP<br>QNNAHRDKEGDDQSHWRYGGDPPWPRVSPACAGRFQSPVDIRPQLAAFCPALRPLELLGFQLPPLPELRLRNNGHSVQLTLPPGLEMALGPGREYRALQLHLHWGAA<br>GRPGSEHTVEGHRFPAEIHVVHLSTAFARVDEALGRPGGLAVLAAFLEEGPEENSAYEQLLSRLEEIAEEGSETQVPGLDISALLPSDFSRYFQYEGSLTTPPCAQGVIWTV<br>FNQTVMLSAKQLHTLSDTLWGPGDSRLQLNFRATQPLNGRVIEASFPAGVDSSPRAAEPVQLNSCLAAGDLPAVPPQQWALSAGNGSSEVEVVPFQEVWGRSYCRA<br>LERLVDVVSEYPSEVEHMFSPSCVSLLRCTGCCGNASLHCVPVETANVTMQLLKIRSGDRPSYVELTFSQHVRCECAGSGSHHHHHHITPPRYRADEGSGSWSHPQFE<br>K*
CA9-PG long with mutPLGF tag and StrepII tag sequence	MKRFLFLLLTISLLVMVQIQTGLSQRLPRMQEDSPLGGGSSGEDDPLGEEDLPSEEDSPREEDPPGEEDLPGEEDLPGEEDLPEVKPKSEEEGSLKLEDLPTVEAPGDPQEP<br>QNNAHRDKEGDDLPAVPPQQWALSAGNGSSEVEVVPFQEVWGRSYCRALERLVDVVSEYPSEVEHMFSPSCVSLLRCTGCCGNASLHCVPVETANVTMQLLKIRSG<br>DRPSYVELTFSQHVRCECAGSGSHHHHHHITPPRYRADEGSGSWSHPQFEK*
CA9-PG short with mutPLGF tag and StrepII tag sequence	MKRFLFLLLTISLLVMVQIQTGLSQRLPRMQEDSPLGGGSSGEDDPLGEEDLPSEEDSPREEDPPGEEDLPGEEDLPGEEDLPEVKPKSEEEGSLKLEDLPAVPPQQWALS<br>AGNGSSEVEVVPFQEVWGRSYCRALERLVDVVSEYPSEVEHMFSPSCVSLLRCTGCCGNASLHCVPVETANVTMQLLKIRSGDRPSYVELTFSQHVRCECAGSGSHH<br>HHHHITPPRYRADEGSGSWSHPQFEK*
CA9-CA with mutPLGF tag and StrepII tag sequence	MKRFLFLLLTISLLVMVQIQTGLSLPTVEAPGDPQEPQNNAHRDKEGDDQSHWRYGGDPPWPRVSPACAGRFQSPVDIRPQLAAFCPALRPLELLGFQLPPLPELRLRN<br>NGHSVQLTLPPGLEMALGPGREYRALQLHLHWGAAGRPGSEHTVEGHRFPAEIHVVHLSTAFARVDEALGRPGGLAVLAAFLEEGPEENSAYEQLLSRLEEIAEEGSETQ<br>VPGLDISALLPSDFSRYFQYEGSLTTPPCAQGVIWTVFNQTVMLSAKQLHTLSDTLWGPGDSRLQLNFRATQPLNGRVIEASFPAGVDSSPRAAEPVQLNSCLAAGDL<br>PAVPPQQWALSAGNGSSEVEVVPFQEVWGRSYCRALERLVDVVSEYPSEVEHMFSPSCVSLLRCTGCCGNASLHCVPVETANVTMQLLKIRSGDRPSYVELTFSQHV<br>RCECAGSGSHHHHHHITPPRYRADEGSGSWSHPQFEK*
CA9-96-137 with mutPLGF tag and StrepII tag sequence	MAPLCPSPWLPLLIPAPAPGLTVQLLLSLLLLVPVHPPEVKPKSEEEGSLKLEDLPTVEAPGDPQEPQNNAHRDKEGDDPAVPPQQWALSAGNGSSEVEVVPFQEVWG<br>RSYCRALERLVDVVSEYPSEVEHMFSPSCVSLLRCTGCCG<br>NASLHCVPVETANVTMQLLKIRSGDRPSYVELTFSQHVRCECAGSGSHHHHHHITPPRYRADEGSGSWSHPQFEK*
CA9-ectodomain (full-length) replacement of 105-119 with GS sequence +Fc(dead)-2E3 tag	MAPLCPSPWLPLLIPAPAPGLTVQLLLSLLLLVPVHPQRLPRMQEDSPLGGGSSGEDDPLGEEDLPSEEDSPREEDPPGEEDLPGEEDLPGEEDLPEVKPKSEEGSGSGSG<br>SGSGSGSGGDPQEPQNNAHRDKEGDDQSHWRYGGDPPWPRVSPACAGRFQSPVDIRPQLAAFCPALRPLELLGFQLPPLPELRLRNNGHSVQLTLPPGLEMALGPG<br>REYRALQLHLHWGAAGRPGSEHTVEGHRFPAEIHVVHLSTAFARVDEALGRPGGLAVLAAFLEEGPEENSAYEQLLSRLEEIAEEGSETQVPGLDISALLPSDFSRYFQYEG<br>SLTTPPCAQGVIWTVFNQTVMLSAKQLHTLSDTLWGPGDSRLQLNFRATQPLNGRVIEASFPAGVDSSPRAAEPVQLNSCLAAGDDKTHTCPPCPAPEAAGGPSVFL<br>FPPKPKDTLMISRTPEVTCVVVDVSHEDPEVKFNWYVDGVEVHNAKTKPREEQYNSAYRVVSVLTVLHQDWLNGKEYKCKVSNKALPAPIEKTISKAKGQPREPQVYTL<br>PPSRDELTKNQVSLTCLVKGFYPSDIAVEWESNGQPENNYKTTPPVLDSDGSFFLYSKLTVDKSRWQQGNVFSCSVMHEALHNHYTQKSLSLSPGGSGSITPPRYRADE<br>*
CA9-96-137 with T115A subsitution mutPLGF tag and StrepII tag sequence	MAPLCPSPWLPLLIPAPAPGLTVQLLLSLLLLVPVHPPEVKPKSEEEGSLKLEDLPAVEAPGDPQEPQNNAHRDKEGDDPAVPPQQWALSAGNGSSEVEVVPFQEVWG<br>RSYCRALERLVDVVSEYPSEVEHMFSPSCVSLLRCTGCCGNASLHCVPVETANVTMQLLKIRSGDRPSYVELTFSQHVRCECAGSGSHHHHHHITPPRYRADEGSGSW<br>SHPQFEK*
CA9-96-137 with 115-117 TVE to NVS subsitution mutPLGF tag and StrepII tag sequence	MAPLCPSPWLPLLIPAPAPGLTVQLLLSLLLLVPVHPPEVKPKSEEEGSLKLEDLPNVSAPGDPQEPQNNAHRDKEGDDPAVPPQQWALSAGNGSSEVEVVPFQEVWG<br>RSYCRALERLVDVVSEYPSEVEHMFSPSCVSLLRCTGCCGNASLHCVPVETANVTMQLLKIRSGDRPSYVELTFSQHVRCECAGSGSHHHHHHITPPRYRADEGSGSW<br>SHPQFEK*
2E3-CA9 (full-length)	MAPLCPSPWLPLLIPAPAPGLTVQLLLSLLLLVPVHPITPPRYRADEGSGSQRLPRMQEDSPLGGGSSGEDDPLGEEDLPSEEDSPREEDPPGEEDLPGEEDLPGEEDLPEV<br>KPKSEEEGSLKLEDLPTVEAPGDPQEPQNNAHRDKEGDDQSHWRYGGDPPWPRVSPACAGRFQSPVDIRPQLAAFCPALRPLELLGFQLPPLPELRLRNNGHSVQLTLP<br>PGLEMALGPGREYRALQLHLHWGAAGRPGSEHTVEGHRFPAEIHVVHLSTAFARVDEALGRPGGLAVLAAFLEEGPEENSAYEQLLSRLEEIAEEGSETQVPGLDISALLP<br>SDFSRYFQYEGSLTTPPCAQGVIWTVFNQTVMLSAKQLHTLSDTLWGPGDSRLQLNFRATQPLNGRVIEASFPAGVDSSPRAAEPVQLNSCLAAGDILALVFGLLFAVT<br>SVAFLVQMRRQHRRGTKGGVSYRPAEVAETGA*
2E3-PDPN (full-length)	MWKVSALLFVLGSASLWVLAEGGSGSITPPRYRADEGSGSASTGQPEDDTETTGLEGGVAMPGAEDDVVTPGTSEDRYKSGLTTLVATSVNSVTGIRIEDLPTSESTVH<br>AQEQSPSATASNVATSHSTEKVDGDTQTTVEKDGLSTVTLVGIIVGVLLAIGFIGAIIVVVMRKMSGRYSP*
2E3-CA9 (full-length) d393-402 (non shed)	MAPLCPSPWLPLLIPAPAPGLTVQLLLSLLLLVPVHPQRLPRMQEDSPLGGGSSGEDDPLGEEDLPSEEDSPREEDPPGEEDLPGEEDLPGEEDLPEVKPKSEEEGSLKLED<br>LPTVEAPGDPQEPQNNAHRDKEGDDQSHWRYGGDPPWPRVSPACAGRFQSPVDIRPQLAAFCPALRPLELLGFQLPPLPELRLRNNGHSVQLTLPPGLEMALGPGRE<br>YRALQLHLHWGAAGRPGSEHTVEGHRFPAEIHVVHLSTAFARVDEALGRPGGLAVLAAFLEEGPEENSAYEQLLSRLEEIAEEGSETQVPGLDISALLPSDFSRYFQYEGSL<br>TTPPCAQGVIWTVFNQTVMLSAKQLHTLSDTLWGPGDSRLQLNFRATQPLNGRVIEASFPAPVQLNSCLAAGDILALVFGLLFAVTSVAFLVQMRRQHRRGTKGGVS<br>YRPAEVAETGA*
2E3-CA9 (full-length) T115A	MAPLCPSPWLPLLIPAPAPGLTVQLLLSLLLLVPVHPQRLPRMQEDSPLGGGSSGEDDPLGEEDLPSEEDSPREEDPPGEEDLPGEEDLPGEEDLPEVKPKSEEEGSLKLED<br>LPAVEAPGDPQEPQNNAHRDKEGDDQSHWRYGGDPPWPRVSPACAGRFQSPVDIRPQLAAFCPALRPLELLGFQLPPLPELRLRNNGHSVQLTLPPGLEMALGPGRE<br>YRALQLHLHWGAAGRPGSEHTVEGHRFPAEIHVVHLSTAFARVDEALGRPGGLAVLAAFLEEGPEENSAYEQLLSRLEEIAEEGSETQVPGLDISALLPSDFSRYFQYEGSL<br>TTPPCAQGVIWTVFNQTVMLSAKQLHTLSDTLWGPGDSRLQLNFRATQPLNGRVIEASFPAGVDSSPRAAEPVQLNSCLAAGDILALVFGLLFAVTSVAFLVQMRRQ<br>HRRGTKGGVSYRPAEVAETGA*
G250 light chain	DIVMTQSQRFMSTTVGDRVSITCKASQNVVSAVAWYQQKPGQSPKLLIYSASNRYTGVPDRFTGSGSGTDFTLTISNMQSEDLADFFCQQYSNYPWTFGGGTKLEIK<br>RTVAAPSVFIFPPSDEQLKSGTASVVCLLNNFYPREAKVQWKVDNALQSGNSQESVTEQDSKDSTYSLSSTLTLSKADYEKHKVYACEVTHQGLSSPVTKSFNRGE*
G250 heavy chain-2E3	DVKLVESGGGLVKLGGSLKLSCAASGFTFSNYYMSWVRQTPEKRLELVAAINSDGGITYYLDTVKGRFTISRDNAKNTLYLQMSSLKSEDTALFYCARHRSGYFSMDYW<br>GQGTSVTVSSASTKGPSVFPLAPSSKSTSGGTAALGCLVKDYFPEPVTVSWNSGALTSGVHTFPAVLQSSGLYSLSSVVTVPSSSLGTQTYICNVNHKPSNTKVDKKV<br>EPKSCDKTHTCPPCPAPEAAGGPSVFLFPPKPKDTLMISRTPEVTCVVVDVSHEDPEVKFNWYVDGVEVHNAKTKPREEQYNSAYRVVSVLTVLHQDWLNGKEYKCKV<br>SNKALPAPIEKTISKAKGQPREPQVYTLPPSRDELTKNQVSLTCLVKGFYPSDIAVEWESNGQPENNYKTTPPVLDSDGSFFLYSKLTVDKSRWQQGNVFSCSVMHEAL<br>HNHYTQKSLSLSPGGSGSITPPRYRADE*
G250 Fab heavy chain-2E3	DVKLVESGGGLVKLGGSLKLSCAASGFTFSNYYMSWVRQTPEKRLELVAAINSDGGITYYLDTVKGRFTISRDNAKNTLYLQMSSLKSEDTALFYCARHRSGYFSMDYW<br>GQGTSVTVSSASTKGPSVFPLAPSSKSTSGGTAALGCLVKDYFPEPVTVSWNSGALTSGVHTFPAVLQSSGLYSLSSVVTVPSSSLGTQTYICNVNHKPSNTKVDKKV<br>EPKSCGSGSITPPRYRADE*
mutPlGF tag control construct	MKRFLFLLLTISLLVMVQIQTGLSHHHHHHLPAVPPQQWALSAGNGSSEVEVVPFQEVWGRSYCRALERLVDVVSEYPSEVEHMFSPSCVSLLRCTGCCGNASLHCV<br>PVETANVTMQLLKIRSGDRPSYVELTFSQHVRCECAGSGSITPPRYRADEGSGS*
Fc(dead) tag control construct	MKRFLFLLLTISLLVMVQIQTGLSHHHHHHGSGSITPPRYRADEGSGSDKTHTCPPCPAPEAAGGPSVFLFPPKPKDTLMISRTPEVTCVVVDVSHEDPEVKFNWYVDGV<br>EVHNAKTKPREEQYNSAYRVVSVLTVLHQDWLNGKEYKCKVSNKALPAPIEKTISKAKGQPREPQVYTLPPSRDELTKNQVSLTCLVKGFYPSDIAVEWESNGQPENNY<br>KTTPPVLDSDGSFFLYSKLTVDKSRWQQGNVFSCSVMHEALHNHYTQKSLSLSPGK*

Protein to cell binding tests

Supplemental Table S2. List of proteins used for binding (competition) experiments

Name	provider	Reference	Host
$CA9^{38-414}$-Fc-avi	acrobiosystems	CA9-H82F5	HEK-293T
$CA9^{38-414}$-His-avi	acrobiosystems	CA9-H82E3	HEK-293T
$PDPN^{23-131}$-His	acrobiosystems	PON-H52H3	HEK-293T
His-$CLEC2^{55-229}$	acrobiosystems	CL2-H5247	HEK-293T
Mouse $CA9^{32-390}$-His	acrobiosystems	CA9-M52H3	HEK-293T

Protein-to-cell binding was detected using Alexa Fluor 488 or 647 conjugated streptavidin (invitrogen, #S11223 or #S21374), anti-CA9 antibody (BD, #568278) or anti-His (BioLegend, #362611).

Binding of 2E3 tagged constructs expressed into conditioned HEK-293T media was detected using either Alexa Fluor 488 or Alexa Fluor 647 conjugated anti-2E3 detection antibodies. Conjugation of fluorophores were performed with rapid labelling kits according to manufacturer's instruction (abcam, #ab236553 or #ab269823). For binding experiments cells were incubated at 4°C overnight unless stated otherwise.

Supplemental Table S3. Antibody list

Name	clone	provider	Reference
Human and mouse IgG anti-2E3 (derived from a proprotein-convertase subtilisin/kexin type 9 (PCSK9))	2E3	Customized, production by Evitria, Switzerland	Kuklik et al. 2021[22]
Anti-CLEC2	219133	BD Biosciences	748137
Mouse IgG2a, κ Isotype Control	G155-178	BD Biosciences	562439
Anti-PDPN	NC-08	BioLegend	337026
Rat IgG2a, λ Isotype Ctrl	G013C12	BioLegend	402307
Anti-CA9	M75	BD Biosciences	568278
Mouse IgG2b, κ Isotype Control	27-35	BD Biosciences	555058
Anti-CD3	SK7	BD Biosciences	563798
Mouse IgG1, k Isotype Control	X40	BD Biosciences	562438
Anti-CD14	HCD14	BioLegend	325612
Mouse IgG1, κ Isotype Ctrl	MOPC-21	BioLegend	400130
Anti-CD66c	B6.2/CD66	BD Biosciences	742688
Mouse IgG1, k Isotype Control	X40	BD Biosciences	563330
Anti-CD56	B159	BD Biosciences	555516
Mouse IgG1, κ Isotype Control	MOPC-31C	BD Biosciences	550617
Anti-CD20	L27 (CE_IVD)	BD Biosciences	335828
Anti-CD19	SJ25C1	BD Biosciences	341113
Mouse IgG1 κ Isotype Control	MOPC-21	BD Biosciences	565573
Anti-mouse CD41	MWReg30	BioLegend	133911
Rat IgG1 κ Isotype Control	RTK2071	BioLegend	400429
Anti-His	J095G46	BioLegend	362611
anti-CD41a	HIP8	BD Biosciences	559777
Mouse IgG1, κ Isotype Control	MOPC-21	BD Biosciences	555751
anti-CD41a	HIP8	BD Biosciences	740975
Mouse IgG1, κ Isotype Control	MOPC-21	BD Biosciences	555748
anti-CD42a	ALMA.16	BD Biosciences	558818
anti-CD42b	HIP1	BD Biosciences	740075
anti-CD62P	AK4	BioLegend	304906
Anti-CD14	HCD14	BioLegend	325610
Mouse IgG1, κ Isotype Control	MOPC-21	BD Biosciences	557702
Calcein Red-Orange	NA	invitrogen	C34851

AlphaLISA

Supplemental Table S4. List of reagents, concentrations and material used for AlphaLISA experiments. Donor beads loaded with $CA9^{38-414}$-His-avi were incubated with 2 µl of conditioned media library protein and anti-2E3 coated protein A acceptor beads (0.5 nM) for 2h at room temperature. Screening assays were performed in 1536-well plates, with a final volume of 3 µL in AlphaLISA Immunoassay Buffer. Confirmation experiments were performed in 384-well plates with a final volume of 10 µL using 7 µL of conditioned media ectodomains. Donor beads were excited at 680 nm and acceptor bead emission was detected at 615 nm using a PheraSTAR FSX plate reader (BMG Labtech).

Name	provider	Reference	Additional information
$CA9^{38-414}$-His-avi	acrobiosystems	CA9-H82E3	Expression system: HEK-293T, final concentration 60 nM
streptavidin AlphaSCREEN donor beads	Revvity	6760002	final concentration 20 ug/mL
Protein A AlphaLISA Acceptor Beads	Revvity	AL101R	final concentration 10 ug/mL
AlphaLISA Immunoassay Buffer Buffer, 5X	Revvity	AL000F	NA
Greiner 384-well microplates (small volume), white	Greiner	784075	final assay volume 10 µL
Greiner 1536-well microplates, white	Greiner	782075	final assay volume 3 µL
Anti-2E3 antibody	Evitria, (CH)	Kuklik et al.[22]	human

Supplemental Table S5. List of recombinant library proteins with binding to CD14^{high} stained cells with a 1.5x greater Median Fluorescence Intensity (MFI) compared to negative controls. Binding could be indirect via platelets (see main text)

protein	MFI normalized to neg. control	protein tag
**LYAM3**	41,420	Fc(dead)-2E3
**GSLG1**	6,911	Fc(dead)-2E3
**CD2**	6,102	Fc(dead)-2E3
**JAM2**	5,065	Fc(dead)-2E3
**TNFA**	4,981	mutPLGF
**PDPN**	4,641	Fc(dead)-2E3
**COL12**	3,622	mutPLGF
**TYRO3**	3,267	Fc(dead)-2E3
**SHPS1**	3,267	Fc(dead)-2E3
**HAVR1**	3,116	Fc(dead)-2E3
**PDIA1**	2,829	Fc(dead)-2E3
**OMGP**	2,557	Fc(dead)-2E3
**CD14**	2,511	Fc(dead)-2E3
**XXLT1**	2,461	mutPLGF
**CAH9**	2,180	Fc(dead)-2E3
**OLR1**	2,079	mutPLGF
**TIMD4**	1,968	Fc(dead)-2E3
**CD6**	1,888	Fc(dead)-2E3
**SARAF**	1,782	Fc(dead)-2E3
**X3CL1**	1,777	Fc(dead)-2E3
**SUSD4**	1,602	Fc(dead)-2E3
**CD47**	1,557	Fc(dead)-2E3
**EXTL1**	1,544	mutPLGF
**FCERA**	1,518	Fc(dead)-2E3
**RENR**	1,513	Fc(dead)-2E3

Supplemental Table S6. CA9^{ectodomain} AlphaLISA read-out. Excitation was determined in duplicate measurement. Each value was divided by the median value of all constructs (set as 0 nM control)

protein	median fold change	protein tag	protein	median fold change	protein tag	protein	median fold change	protein tag
**GLT16**	958,72	mutPLGF	CNTN4	15,29	Fc(dead)-2E3	LRRT2	5,35	Fc(dead)-2E3
**CLC1B**	866,14	mutPLGF	VSTM4	14,33	Fc(dead)-2E3	OMGP	4,99	Fc(dead)-2E3
**LYAM3**	665,90	Fc(dead)-2E3	MUCL3	14,21	Fc(dead)-2E3	EPGN	4,96	Fc(dead)-2E3
**CR1**	269,08	Fc(dead)-2E3	LRIG1	12,85	Fc(dead)-2E3	TNF12	4,58	mutPLGF
**CR2**	236,76	Fc(dead)-2E3	BMR1A	11,60	Fc(dead)-2E3	SACA4	4,28	Fc(dead)-2E3
**TIMD4**	114,85	Fc(dead)-2E3	PXYP1	10,81	mutPLGF	MUSK	4,18	Fc(dead)-2E3
**ICAM5**	112,13	Fc(dead)-2E3	GSLG1	10,39	Fc(dead)-2E3	ERAP1	4,13	mutPLGF
**GLCE**	79,19	mutPLGF	PRTG	10,30	Fc(dead)-2E3	C163A	4,12	Fc(dead)-2E3
**MDGA1**	76,97	Fc(dead)-2E3	TIE1	10,08	Fc(dead)-2E3	ADA18	3,94	Fc(dead)-2E3
**NFASC**	73,63	Fc(dead)-2E3	CHSTB	9,71	mutPLGF	MA1C1	3,73	mutPLGF
**CNTN5**	70,58	Fc(dead)-2E3	IGDC4	9,62	Fc(dead)-2E3	CDON	3,66	Fc(dead)-2E3
**GFRA2**	49,74	Fc(dead)-2E3	TX101	8,74	Fc(dead)-2E3	FICD	3,60	mutPLGF
**TRML2**	34,81	Fc(dead)-2E3	TRIL	8,25	Fc(dead)-2E3	C1C1L	3,60	mutPLGF
**TM11F**	31,57	mutPLGF	CSF1R	7,82	Fc(dead)-2E3	PCDH8	3,45	Fc(dead)-2E3
**NLGN1**	29,85	Fc(dead)-2E3	APCD1	6,67	Fc(dead)-2E3	MSRE	3,43	mutPLGF
**B4GN1**	26,25	mutPLGF	I20RA	6,31	Fc(dead)-2E3	MUC13	3,43	Fc(dead)-2E3
**PTPRD**	25,50	Fc(dead)-2E3	MAG	5,78	Fc(dead)-2E3	ECEL1	3,41	mutPLGF
**NLGN2**	17,53	Fc(dead)-2E3	SIA8C	5,69	mutPLGF	DJC10	3,35	Fc(dead)-2E3
**AGRG1**	15,52	Fc(dead)-2E3	CS018	5,38	Fc(dead)-2E3	CD109	3,34	Fc(dead)-2E3

Supplemental Table S7. Lead variants of GALNT16 (sorted by p-Value) from different genome wide association studies sourced from Open Target Genetics [75,76]

Supplementary Figures

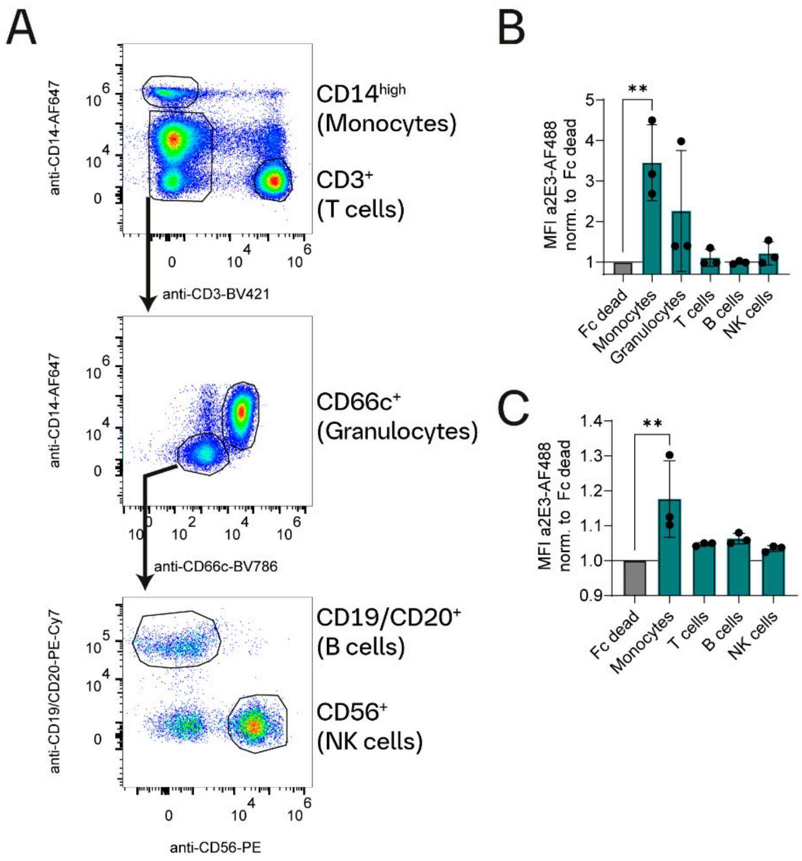

Supplemental Figure S1. CA9 binding is detected in whole blood samples to CD14^{high} expressing cells. (A) Example for gating strategy to distinguish different cell types in whole blood samples after red blood cell lysis. (B) CA9 binding test to leukocytes from peripheral blood after red blood cell lysis with gating as shown in B. Binding was tested to CD14^{high} (monocytes), CD3^{high} (T cells), CD66c^{high} (granulocytes), CD56^{high} (NK cells) and CD19/20^{high} B cells. Each dot represents one donor. CA9 binding is detected to CD14^{high} cells. **=0.0061 (ordinary one-way ANOVA combined with Dunnett's multiple comparison test to negative control (2E3-Fc(dead) tag only). (C) CA9 binding test to leukocytes from peripheral blood after PBMC isolation. Binding was tested to CD14^{high} (monocytes), CD3^{high} (T cells), CD56^{high} (NK cells) and CD19/20^{high} B cells. Each dot represents one donor. CA9 binding is detected to CD14^{high} cells. **=0.0048 (ordinary one-way ANOVA combined with Dunnett's multiple comparison test to negative control (2E3-Fc(dead) tag control).

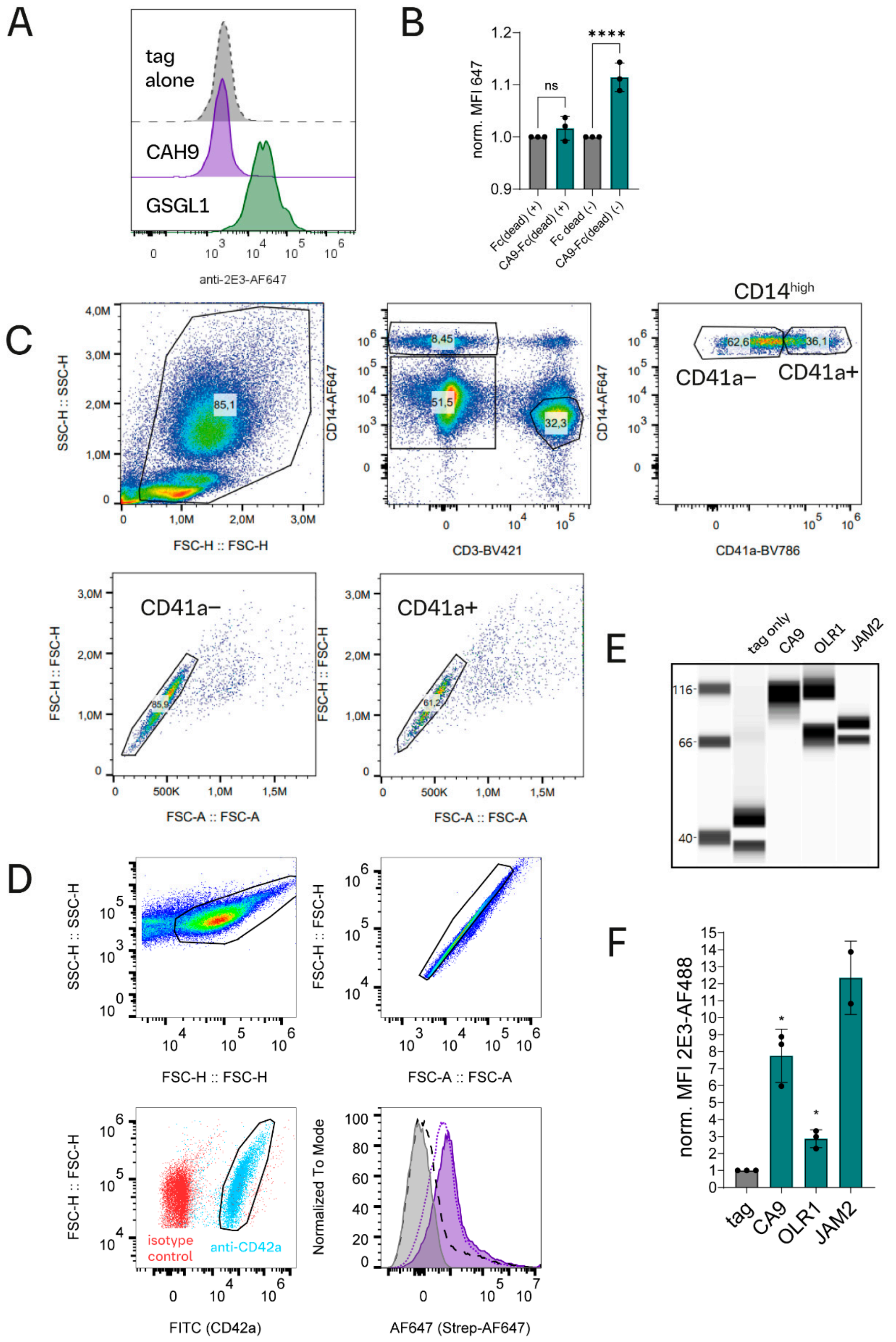

Supplemental Figure S2. CA9 binds to platelets. (A) CA9-Fc(dead)^{2E3} binding test to THP-1 cell line. THP-1 cells incubated with library protein constructs (GSGL1-Fc(dead)^{2E3} (green solid line) or CA9-Fc(dead)^{2E3} (purple solid line)) or tag control protein (grey dashed line) are stained with anti-2E3-Alexa Fluor 647. Binding is observed for tagged GSGL1 but not for CA9. (B) CA9-Fc(dead)-2E3 binding to Monocytes isolated in presence (+) or absence (-) of a platelet removal cocktail (STEMCELL™). Each dot represents

one donor. Only in absence of platelet removal cocktail binding of tagged ectodomain of CA9 is detected to CD14+ cell (****<0.0001; ordinary one-way ANOVA with Šídák's multiple comparisons test). **c**, Example of gating used to distinguish platelet free (CD41a−) and platelet associated CD14^{high} cells used for quantification in main Figure 2B. (D) Top and bottom left panels: Example of gating strategy for buffy-coat derived platelets used in main Figure 2C-D. bottom right: Histograms of Alexa Fluor (AF) 647 of anti-CD42a-FITC stained platelets. solid line (grey filling): Fluorescence minus one control (FMO) for AF647, dashed black line (no filling): Streptavidin-AF647 (Strep) staining without protein addition, dotted purple line (no filling): Strep-AF647 with human CA9$^{38\text{-}414}$-His-avi, solid line (purple filling): Strep-AF647 with human CA9$^{38\text{-}414}$-Fc-avi. (E) Simple protein immunoassay analysis to assess expression of CA9, OLR1, JAM2 library constructs or a tag control construct. Detection with anti-2E3 antibody. (F) Binding test of indicated library constructs to Calcein$^{Red\text{-}Orange}$ stained buffy-coat derived platelets. Quantification of MFI fold change compared to tag control constructs. Statistics: *=0.027 (CA9) *=0.0394 (OLR1); one-way ANOVA, Dunnett's multiple comparison test. Each dot represents one donor.

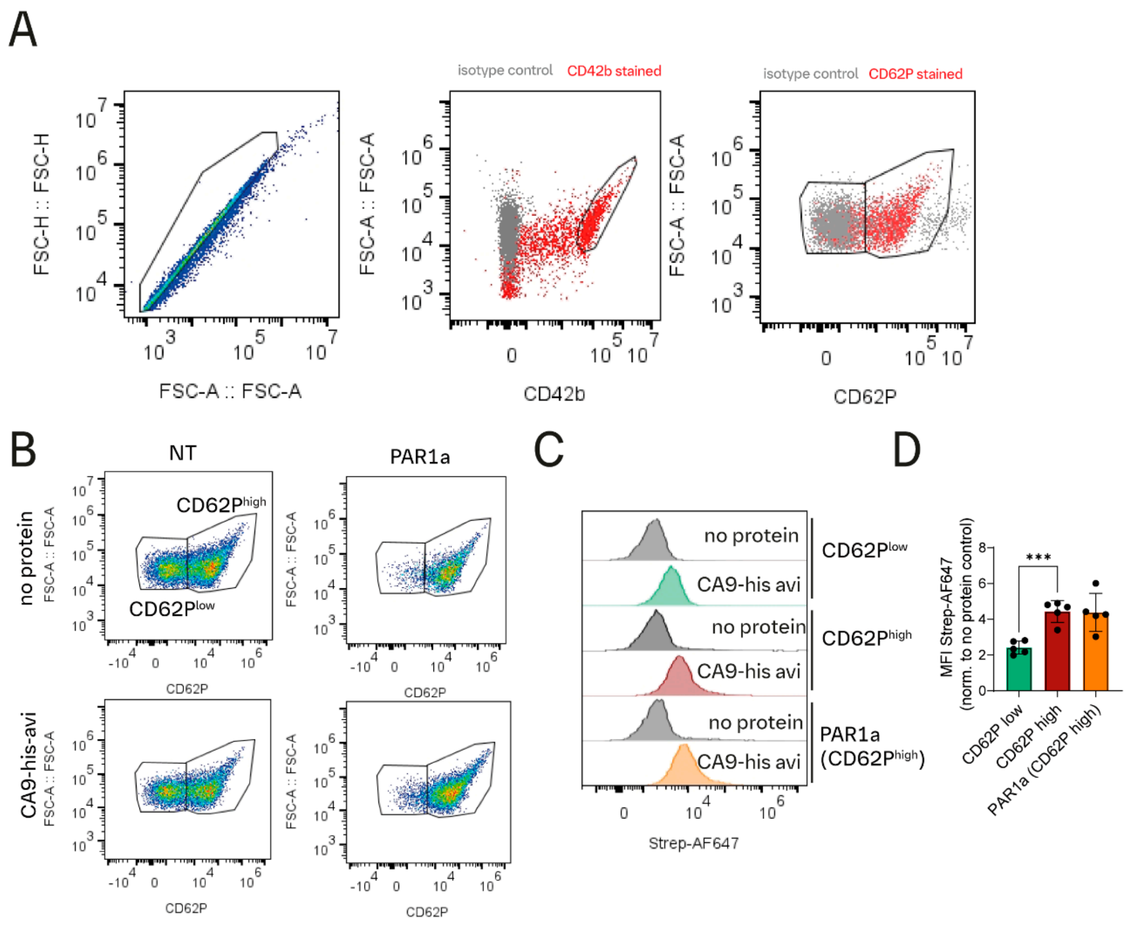

Supplemental Figure S3. CA9 binds to inactive and CD62P+ human platelets. (A) Example of gating strategy for differentiation of CD62P$^{high/low}$ platelets in flow cytometry of buffy coat-derived platelets. (B) Example of CD62P^{high} and CD62P^{low} mixed population of buffy coat-derived platelets incubated with soluble biotinylated 4,45 ng/ml CA9$^{38\text{-}414}$-His-avi overnight at 37°C. PAR1 agonist (PAR1a) (8 µM TRAP-6) treatment shifts population towards CD62P^{high} expression. (C) Histograms of CD62 high or low untreated or PAR1a treated human platelets incubated with or without biotinylated CA9$^{38\text{-}414}$-His-avi. CA9 binding is detected with Alexa Fluor 647 coupled Streptavidin. Improved binding is observed for CD62P^{high} compared to CD62P^{low} platelets. (D) Streptavidin-Alexa Fluor 647 MFI quantification. Graph shows fold changes for each donor relative to untreated control. Each dot represents one donor. Statistics show unpaired t-test (***=0.002).

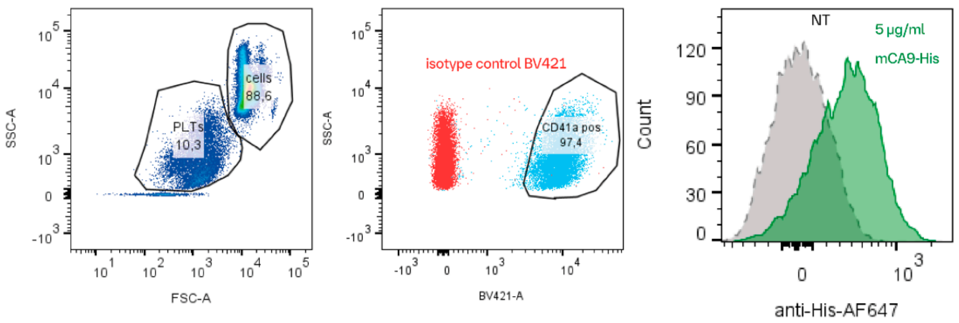

Supplemental Figure S4. CA9 binding to platelets is conserved in mouse. Mouse CA9 binds to platelets from mouse blood samples. Left: cell size-based gating strategy to distinguish platelets and other cells in diluted mouse blood. Middle: CD41 stained platelets of size gated platelets compared to isotype control. Right: Binding of mouse CA9-His (green solid line, 5 µg/ml protein was incubated for 30 min at room temperature in presence of 2.5 mM Gly-Pro-Arg-Pro) compared to untreated control (grey dashed line) to size and CD41⁺ gated platelets of mouse.

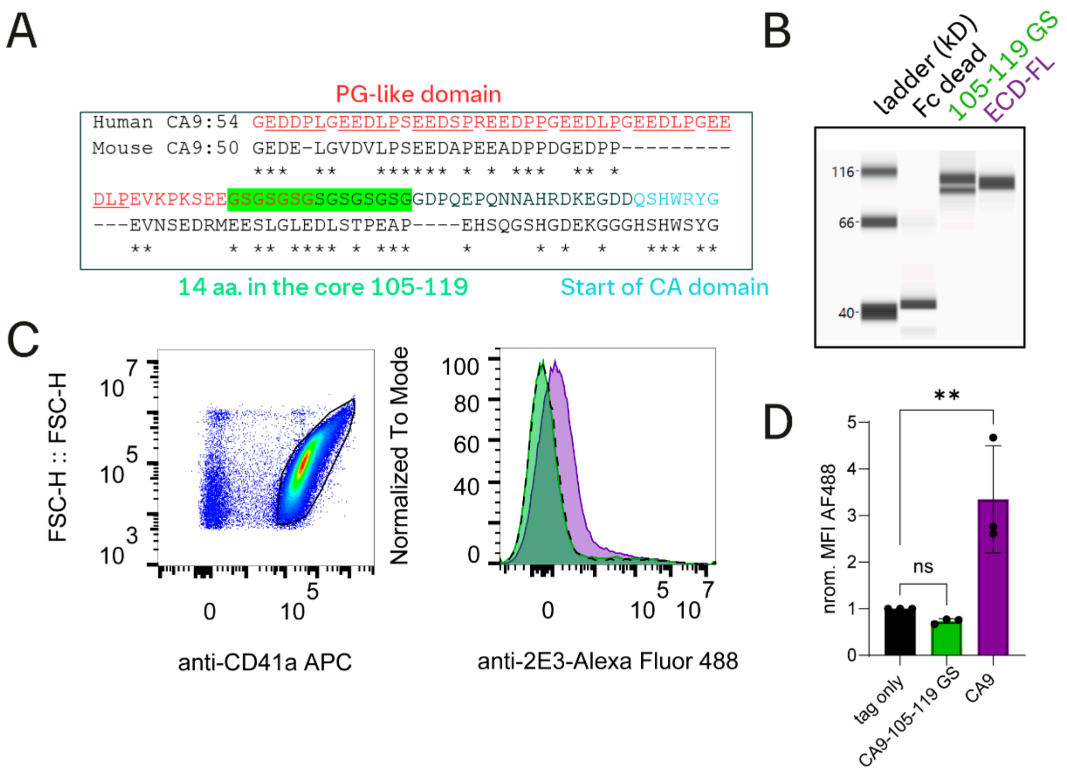

Supplemental Figure S5. CA9 binding is ablated in absence of a 14 amino acid core region within the platelet binding region. (A) Schematic representation of highly conserved platelet binding core region. Sequence alignment between mouse and human CA9. Stars (*) below sequence indicate amino acid conservation with original human CA9 sequence. A 14-amino-acid core sequence (highlighted in green) with high sequence conservation has been replaced with a GS sequence. (B) Simple protein immunoassay analysis to assess expression of CA9 or tag control construct. Detection with anti-2E3 antibody. (C) Example of binding test of CA9 constructs to buffy-coat derived platelets. Left: Gating of CD41a stained cells. Right: Histogram of 2E3 stained cells. Cells were incubated with conditioned media of HEK-293T cell transfected with a tag control (black dashed line) or CA9 full-length ectodomain wild-type (purple solid line) or a CA9 full-length ectodomain construct with a glycine-serine substitution of amino acids 105-119 (green solid line). (D) Binding test of indicated protein constructs to buffy-coat derived platelets.

Quantification of MFI fold change compared to tag control constructs. Statistics: **=0.0088; one-way ANOVA, Dunnett's multiple comparison test. Each dot represents one donor.

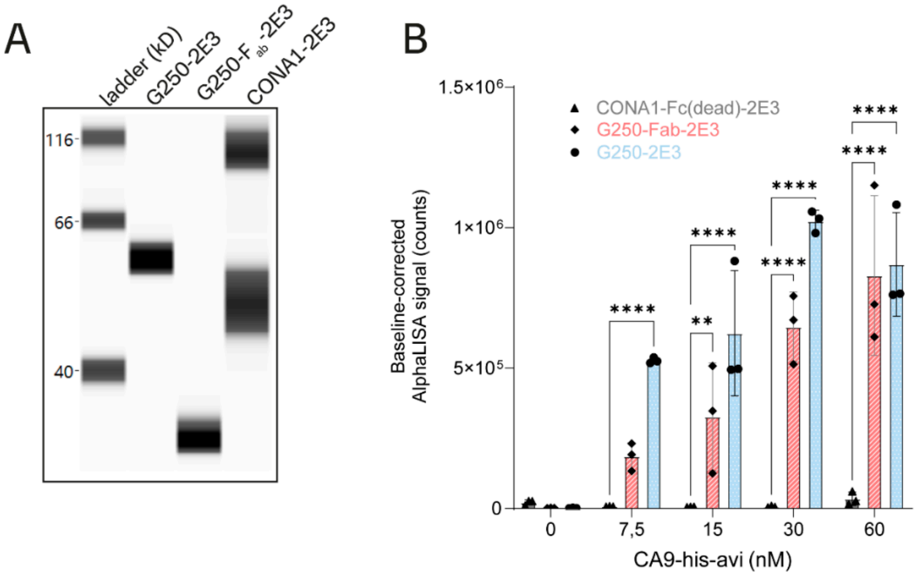

Supplemental Figure S6. Detection of CA9-Girentuximab interaction with AlphaLISA setup. (A) Protein expression test in supernatants of HEK-293T cells 3 days post transfection by anti-2E3 immunoassay. (B) CA9 AlphaLISA validation assay. $CA9^{38-414}$-His-avi coated beads are mixed with HEK-293T supernatants expressing constructs shown in A. Emission signals are measured and compared. For both G250 constructs strong signal increases were detected but not for a negative control (CONA1) of our protein library. One-way ANOVA with Tukey's multiple comparison test (****<0.0001, **=0.0081)

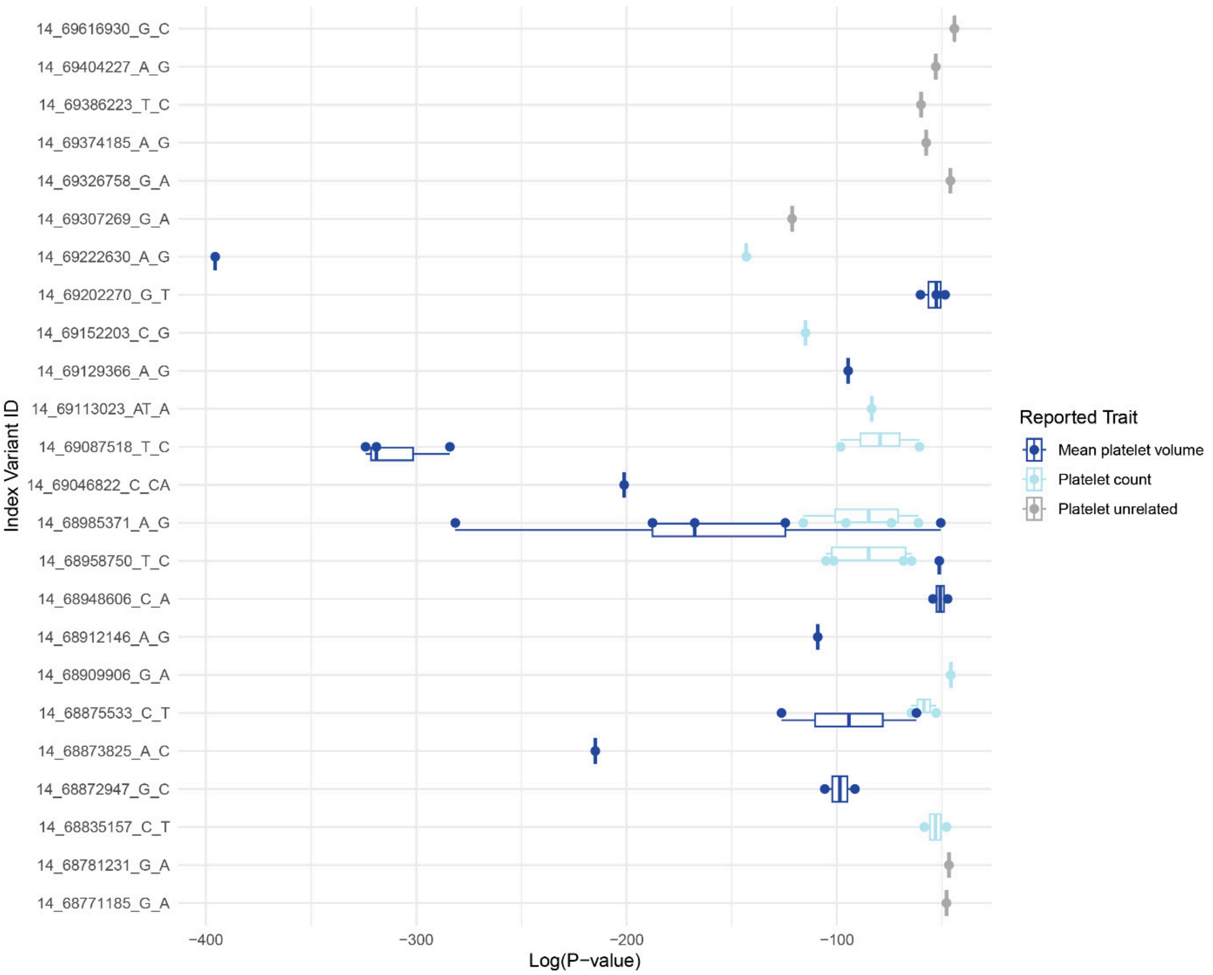

Supplemental Figure S7. Platelet-related traits associate with UDP-N-α-D-galactosamine polypeptide N-acetylgalactosaminyltransferase 16 (GALNT16/GLT16) gene in genome wide association studies. Graphical representation of the 50 most significant variant-gene-trait associations of the *GALNT16* gene identified in a comparative analysis of several Genome-Wide Association Studies (GWAS). Data sourced from Open Targets Genetics platform[75, 76] and plotted using RStudio.
